# Presence of Interferon Regulatory Factor-1 in Aggressive and Nonaggressive Histological Variants of Basal Cell Carcinoma Specimens

**DOI:** 10.4103/0974-2077.63395

**Published:** 2010

**Authors:** Nurten Turhan-Haktanir, F Husniye Dilek, Yavuz Demir, Onder Sahin

**Affiliations:** *Faculty of Medicine, Department of Plastic, Reconstructive, and Aesthetic Surgery, Afyonkarahisar Kocatepe University, Turkey*; 1 *Department of Pathology, Afyonkarahisar Kocatepe University, Turkey*

**Keywords:** Basal cell carcinoma, interferon, interferon regulatory factor-1

## Abstract

**Background:**

Expression of Interferon regulatory factor-1 (IRF-1) has been demonstrated in a variety of cancers previously. To the best of our knowledge, there is only one report about the IRF-1 expression in basal cell carcinoma (BCC) specimens, which has demonstrated increased expression of the IRF-1 gene in BCC versus normal skin. Furthermore, IRF-1 expression has not been compared between aggressive and nonaggressive subtypes of BCC before.

**Aims:**

Our aim is to examine the relation between IRF-1 staining patterns of aggressive and nonaggressive histological subtypes of BCC.

**Materials and Methods:**

Thirty-seven archival tissue specimens from 33 BCC patients were stained for cytoplasmic and nuclear IRF-1. Twenty-five tumors were grouped as nonaggressive and 12 were grouped as aggressive histological variants, according to the established criteria.

**Results:**

IRF-1 was not only expressed in tumor cells, but also in some surrounding inflammatory cells. There was no significant difference between the groups for cytoplasmic or nuclear IRF-1 staining. Aggressive or nonaggressive histological subtypes did not show any statistical difference for gender, relapse, treatment method, or localization. When staining was compared with gender, relapse, treatment method, localization, and tumor grades, no significant difference was found.

**Conclusions:**

Interferon seems to be expressed in BCCs with both aggressive and non aggressive histology. Further studies are needed to elaborate the precise diagnostic and prognostic roles and utility of interferon in the treatment of BCC.

## INTRODUCTION

Basal cell carcinoma (BCC) is a common cutaneous malignancy, and its incidence has been estimated to have increased from 20 to 80% worldwide over the last three decades.[1] Increasing incidence and high risk of new tumors in BCC patients, make the management of this tumor more important. Preferred treatment of choice is surgery; however, various treatment modalities including electrodessication, radiotherapy, cryotherapy, Mohs micrographic surgery, carbon dioxide laser, topical 5-fluorouracil, photodynamic therapy, and interferons have been used.[2]

Interferons (IFNs) are members of a family of proteins secreted naturally by human cells in response to viral infection and a variety of synthetic and biological inducers. Recombinant interferon α-2b has been used in the treatment of various viral infections and tumors.[3–11] Earlier, interferon treatment had been studied only in low-risk or nonaggressive BCCs (nodular and superficial). In nodular and superficial type BCCs, injection of interferon-α-2b has been reported with comparable cure rates.[9,12,13] However, treatment of BCC with intra lesional interferon is still essentially investigational and is unlikely to prove useful in high-risk tumors.[14]

In view of this, a study on the expression of Interferon regulatory factors (IRFs) may be beneficial in elucidating the role of IFNs in the management of BCC. IRFs constitute a family of nine mammalian transcription factors (IRF-1 to -9). The first discovered member of this family, IRF-1, has a remarkable functional diversity in the regulation of cellular responses in the host's defence. It targets different sets of genes, in various cell types, in response to diverse cellular stimuli and evokes appropriate innate and adaptive immune responses.[15] IRF-1, which acts as the effector arm of the IFN-γ response in tumor cells, is a transcriptional activator of gene expression. In a variety of cancers, the expression of IRF-1 has been demonstrated.[16–28] However, to the best of our knowledge, there is only one previous report about the IRF-1 expression in BCC specimens, despite the reported use of interferon in BCC patients. Kaporis *et al.*, showed increased expression of the IRF-1 gene in BCC versus normal skin, by microarray analysis.[19] The authors have not compared the IRF-1 expression between the aggressive and nonaggressive subtypes of BCC.

The purpose of the present study is to examine the correlation between the IRF-1 staining patterns in aggressive and nonaggressive histological subtypes of BCC.

## MATERIALS AND METHODS

Thirty-seven formalin-fixed, paraffin-embedded, archival tissue specimens from 33 patients with BCC were cut into 4 μm sections. The tissue sections were deparaffinised in xylene and dehydrated in a graded series of ethanol. Sections were placed in 0.01 M citrate buffer at pH 6.0 and heated in a microwave oven for 20 minutes, to perform epitope retrieval. Endogenous peroxidase was quenched with 3% hydrogen peroxide; the samples were rinsed thrice for 5 minutes each time in Tris-buffered saline (TBS). Thereafter, the tissue sections were incubated with 1:100 rabbit antihuman IRF-1 polyclonal antibody M-20 (sc-640, Santa Cruz Biotechnology, Santa Cruz, CA) overnight, at 4°C. After being washed in TBS thrice, the UltraVision biotinylated polyvalent IgG30 and streptavidin horseradish peroxidase (Lab Vision, Fremont, USA) were used for the subsequent steps, according to the manufacturer's instructions. Chromogenic development was accomplished by using aminoethylcarbazol. The slides were then slightly counterstained with Mayer's hematoxylin, dehydrated, and applied to coverslips. Negative controls were included in each experiment by incubating the tissue sections with an antibody dilution buffer instead of the primary antibody. Positive control slides consisted of mouse uterus tissues. Cytoplasmic or nuclear staining for IRF-1 was considered positive only when more than 5% of the tumor cells in the entire tumor area were judged to be positive. The histological variants of BCC were defined according to the World Health Organisation (WHO) classification of tumors.[20] The study protocol was approved by the institutional Ethics Committee.

Chi-square and Fisher's exact tests were used for statistical analysis. The significance was defined as *P* < 0.05.

## RESULTS

The characteristics of patients are presented in Table 1 with percentages. The most frequent subtype was nodular, followed by mixed, infiltrative, nodular cystic, BCC with adnexal differentiation, keratotic, nodular pigmented, nodular cribriform, and superficial BCCs. All mixed types were composed of nodular and infiltrative components. Nodular, nodular cystic, nodular pigmented, nodular cribriform, keratotic, BCC with adnexal differentiation, and superficial subtypes were grouped as nonaggressive, and the others (infiltrative and mixed) were grouped as aggressive, according to the previous literature.[20,21] Thus, 25 tumors (67.6%) were grouped as nonaggressive and 12 tumors (32.4%) were grouped as aggressive histological variants of BCC. No metastasis was present in the patients. Six of the tumors had relapsed after surgical excision.

**Table 1:** Characteristics of basal cell carcinoma patients

Characteristics	Total number with characteristic (n)	Value in percentage (%)
Age at presentation (y)		
Median	61.7 ± 14.1	-
Range	36 – 87	-
Gender		
Male	15	45.5
Female	18	54.5
Tumor localization		
Nose	14	37.8
Scalp	8	21.6
Canthal area	8	21.6
Cheek	6	16.2
Trunk	1	2.7
Pathological classification		
Nonaggressive subtypes	25	67.6
Nodular	17	45.9
Nodular cystic	2	5.4
Adnexal differentiation	2	5.4
Keratotic	1	2.7
Nodular pigmented	1	2.7
Nodular cribriform	1	2.7
Superficial	1	2.7
Aggressive subtypes	12	32.4
Infiltrative	2	5.4
Mixed (infiltrative and nodular)	10	27.0
Applied treatment		
None	2	5.4
Primary suturing	16	43.2
Grafting	9	24.3
Flap reconstruction	10	27.0
Tumor grades		
T1/S1	32	86.5
T2/S2	2	5.4
T3/S2	2	5.4
T4/S3	1	2.7

**Figure 1:**
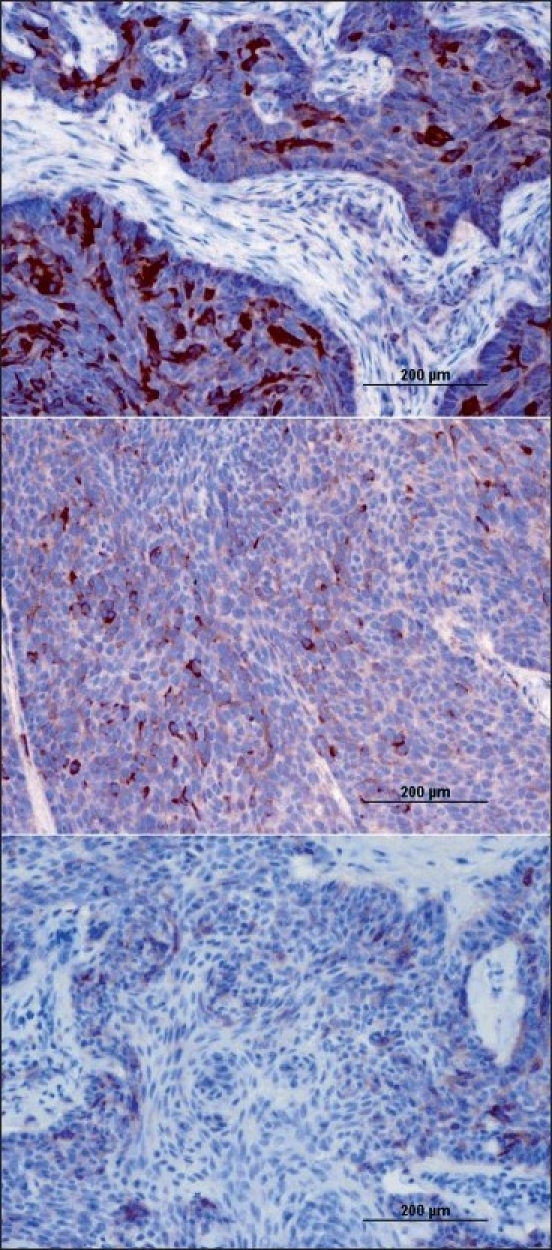
IRF-1 protein expression in representative basal cell carcinomas: strong cytoplasmic staining (upper), moderate cytoplasmic staining (middle), and weak cytoplasmic staining (lower) (× 200)

**Figure 2:**
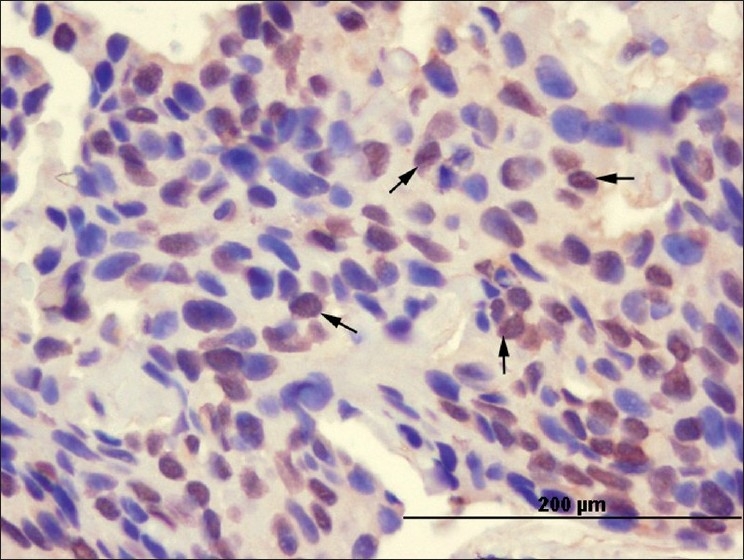
Basal cell carcinoma cells showing nuclear staining pattern (arrows) with the anti-IRF-1 antibody (× 400)

IRF-1 staining was both cytoplasmic and nuclear [Figures 1 and 2]. IRF-1 was not only expressed in the tumor cells, but also in some inflammatory cells in the surrounding stroma. Cytoplasmic expression of IRF-1 was not homogeneous. It varied in its distribution in the tumors. Results of the cytoplasmic and nuclear staining with IRF-1 are presented in Table 2.

**Table 2:** IRF-1 staining in aggressive and nonaggressive histological variants of basal cell carcinoma

Histological variant of tumors	IRF-1* positive		IRF-1* negative		Total
			
	Cytoplasmic	Nuclear	Cytoplasmic	Nuclear
Nonaggressive	19 (65.5)	20 (66.6)	6 (75.0)	5 (71.4)	25
Aggressive	10 (34.5)	10 (33.3)	2 (25.0)	2 (28.6)	12
Total	29 (100)	30 (100)	8 (100)	7 (100)	37

* IRF: interferon regulatory factor, Figures in parenthesis are in percentage

There were no significant differences between aggressive and nonaggressive groups for cytoplasmic or nuclear IRF-1 staining (*P* = 0.811) [Table 2].

Aggressive or nonaggressive histological subtypes did not show any statistical difference for gender, relapse, treatment method, or localization (*P* > 0.05). When staining was compared with gender, relapse, treatment method, localization, and tumor grades, no significant difference was found (*P* > 0.05).

## DISCUSSION

Interferon regulatory factor-1 is a DNA-binding factor that functions as a regulator of both type I interferon (interferon-α and β) and interferon-inducible genes.[14] IFN-γ has also been found to be a potent inducer of IRF-1, mediating many IFN-γ effects on cells.[22–24] Besides, IRF-1 has direct antiproliferative effects, thus acting as a tumor suppressor and tumor susceptibility gene.[25] The expression of IRF-1 has been reported in various cancers.[16–18] Since 1986, interferon injections have been used for the treatment of nodular and superficial BCCs.[9] However, to the best of our knowledge, reports of interferon use in aggressive types of BCC are very limited in the English-language literature.[26,27] Kaporis *et al.* showed increased expression of the IRF-1 gene in BCC versus normal skin, by microarray analysis.[19]

The ideal subtyping of BCCs must be able to correlate with the clinical behaviour and treatment requirements. However, such a classification is yet to be defined. Currently, the most preferred classification is based predominantly on the histological growth pattern. This classification contributes to the useful concept of low- and high-risk histological subtypes of BCC. High-risk BCCs are characterized by one or more of the following: an increased probability of subclinical extension, incomplete excision, aggressive local invasive behaviour, and local recurrence.[28] Aggressive BCCs (multifocal, deeply infiltrating, or showing an aggressive growth pattern) may be therapeutically challenging and treatment can be difficult and cosmetically disfiguring. As they involve considerable tissue loss and may demand a radical surgery, alternative and adjunctive treatment methods are frequently required. As previously mentioned, we have found very limited reports regarding interferon use in the aggressive subtypes of BCC, in literature: Stenquist *et al.*, reported the efficacy of intra-lesional interferon in the treatment of aggressive BCCs in 15 patients with histologically proven primary morpheaform or recurrent BCCs, who had been referred for Mohs surgery, and they concluded that interferon was able to cure only a minority of the aggressive BCCs.[27] On the other hand, Fenton *et al.*, used this treatment for 11 patients with clinically aggressive BCC of the eyelids.[26] Three of their patients had morpheaform type BCC. None of their patients showed any residual tumor, except one who had developed a BCC on the outer lid, as a new primary site.

In the present study, IRF-1 expression was observed in 37 BCC specimens that were grouped as having aggressive and nonaggressive histological characteristics. Not making a comparison of the normal skin tissue from BCC patients with the tumor specimens for IRF-1 seems to be a limitation of our study. Such an evaluation would reinforce the conclusions of the study. Another drawback of the study is the limited number of samples analyzed. A study with a major number of patients is necessary.

While we made a comparison between groups with aggressive and nonaggressive histological characteristics, no significant difference of IRF-1 expression was found. IRF-1 expression was found in BCC of all types, both aggressive and nonaggressive. However, the clinical implications of this finding and its possible relation to the use of IFNs need to be studied further in specific trials.
